# Dilatação Aneurismática Isolada do Átrio Direito, com Resolução Cirúrgica, em Adulta de 53 Anos

**DOI:** 10.36660/abc.20220870

**Published:** 2023-06-07

**Authors:** Edmar Atik, Alessandra Costa Barreto, Maria Angélica Binotto, Marcelo B. Jatene

**Affiliations:** 1 Hospital das Clínicas Faculdade de Medicina Universidade de São Paulo São Paulo SP Brasil Instituto do Coração do Hospital das Clínicas da Faculdade de Medicina da Universidade de São Paulo, São Paulo, SP – Brasil

**Keywords:** Anormalidades Congênitas/cirurgia, Aneurisma Cardíaco, Arritmias Cardíacas, Cardiomegalia, Anomalia de Ebstein/cirurgia, Derrame Pericárdico, Aneurisma Isolado do Átrio Direito

## Introdução

A dilatação aneurismática isolada do átrio direito é uma anomalia congênita rara. Sua identificação ocorre em vários grupos etários, desde o feto até a idade adulta. A manifestação clínica dessa anomalia é variada, conforme a magnitude da dilatação, desde pacientes assintomáticos, em geral em crianças e jovens, até presença de arritmias supraventriculares de difícil controle, insuficiência cardíaca direita, obstrução de vias aéreas, pneumonia e eventos tromboembólicos, em adultos. O diagnóstico é estabelecido após verificação de cardiomegalia em exames rotineiros radiográficos e confirmado posteriormente por ecocardiograma. Chama a atenção nesta patologia o tamanho desproporcional do átrio direito em relação às outras cavidades cardíacas, estando o anel tricúspide também dilatado, mas com valva tricúspide conservada. A insuficiência tricúspide que acompanha o quadro decorre assim da dilatação do anel valvar. A recomendação da correção cirúrgica daí passa a ser profilática na idade infantil dada a boa evolução verificada posteriormente.

O diagnóstico diferencial engloba a anomalia de Ebstein, o derrame pericárdico e tumores torácicos mediastinais.

O tratamento antiarrítmico e a medicação antitrombótica, representada por AAS, geralmente iniciam a conduta clínica, dada a presença de arritmias supraventriculares e de trombos cavitários, mais encontrados na idade adulta.^1 - 12^ A cirurgia de ressecção atrial, idealizada inicialmente por Morrow e Behrendt em 1968,^13^ foi orientada aos pacientes sintomáticos. O bom resultado cirúrgico foi atestado por esses autores em mulher de 23 anos, com flutter atrial e cardiomegalia, com aneurisma do átrio direito. Verificou-se nesta paciente boa evolução, em ritmo sinusal e sem sintomas após a correção efetuada. Desde esse início, tornou-se preconizada a conduta cirúrgica, tendo se estendido mesmo a pacientes assintomáticos, como procedimento eletivo e preventivo das complicações conhecidas.^1 , 2^ Seu diagnóstico durante a vida fetal tem orientado a conduta cirúrgica eletiva nos primeiros anos de vida.^2 , 3^ A evolução longa descrita em alguns casos não operados, com 69 e 88 anos de idade, não expressa benignidade em geral, como observado nesta doença com muitas complicações evolutivas.^4 , 5^

Poucos casos têm sido operados na idade adulta mais avançada,^12^ razão que nos motivou a demonstrar neste relato, em paciente feminina operada com 53 anos de idade.

## Descrição do caso

Dados clínicos: paciente de 53 anos relatava há 19 anos (a partir de 34 anos) crises de palpitações taquicárdicas, quando foi iniciado uso de amiodarona e diltiazen. Há 3 anos, cansaço progressivo e barriga inchada, com melhora após introdução de espironolactona, furosemida, atenolol e enalapril, além de warfarina e omeprazol.

Nesta época, o exame físico revelava bom estado geral, corada, hidratada, acianótica, Peso: 52,00 Kg, Altura:148 cm, IMC: 23,74, PA:100/65 mm Hg, FC: 78 bpm, em ritmo irregular.

Precórdio: impulsões nítidas na borda external esquerda com sopro sistólico na área tricúspide, 2+/6, rude, com irradiação à linha axilar direita. As bulhas eram hipofonéticas. Fígado a 3 cm do rebordo costal direito. Pulmões limpos.

### Exames Complementares

**Figura 1:**
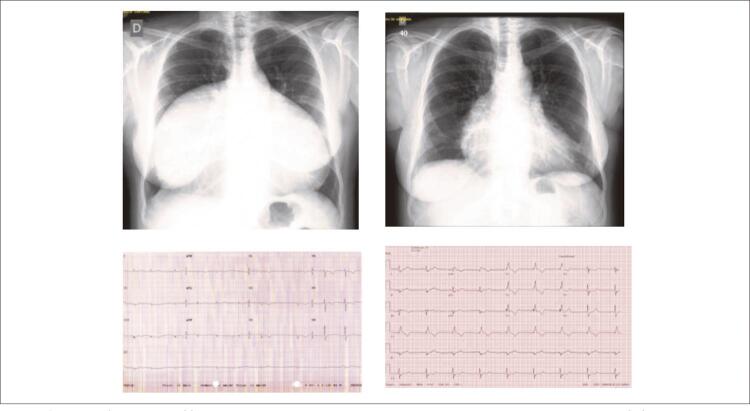
– Radiografias de tórax e ECG´s salientam a área cardíaca exageradamente aumentada com baixa voltagem do complexo QRS em período prévio à cirurgia da dilatação aneurismática do átrio direito à esquerda em contraste com os achados do pós-operatório a longo prazo à direita, com diminuição da cardiomegalia e aumento dos potenciais elétricos.

**Eletrocardiograma:** Ritmo irregular em fibrilação atrial, com frequência cardíaca de 67 bpm, complexo QRS em baixa voltagem no plano frontal com amplitude máxima de 2 mm em todos os complexos. Baixa voltagem nas derivações também do plano horizontal com complexo qs em V1 e ondas S espessadas de V4 a V6, e complexo Rs em V6. Alterações difusas da repolarização ventricular com onda T negativa de V1 a V5. AQRS: -10^o^, e AT: 0^o^ ( Figura 1 ).

**Radiografia de tórax:** Aumento acentuado da área cardíaca com arcos direito e esquerdo que se aproximam do quadril costal bilateral com índice cardiotorácico de 0,90. A trama vascular pulmonar era diminuída bilateralmente ( Figura 1 ).

**Ecocardiograma:** Mostrou acentuada dilatação do átrio direito em grande desproporção com as demais cavidades. As medidas principais corresponderam a: valva mitral: 25 mm, Valva tricúspide: 35, valva pulmonar: 21, valva aórtica: 14, APD: 21, APE: 20, AE: 33, Ao: 28, S=PP: 7, FEVE: 58%.

**Figura 2:**
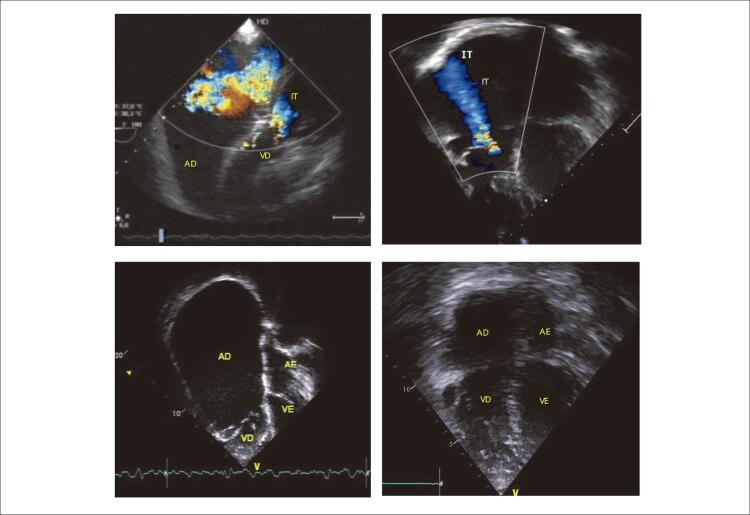
– Ecocardiogramas, em períodos prévios à esquerda e posteriores à cirurgia à direita salientam o acentuado aumento do átrio direito em contraste nítido com as demais cavidades cardíacas em presença da insuficiência tricúspide. Esses elementos quase se normalizaram nas imagens posteriores à intervenção cirúrgica à direita. VD: ventrículo direito; VE: ventrículo esquerdo; AD: átrio direito; AE: átrio esquerdo; IT: insuficiência tricúspide.

As conexões atrioventricular e ventrículo-arterial eram concordantes. Forame Oval Patente com *shunt* esquerda-direita e íntegro o septo ventricular. A valva tricúspide apresentava-se em anel valvar dilatado e com alteração da sua disposição devido à dilatação aneurismática do átrio direito. As cúspides eram discretamente espessadas, com falha de coaptação central e sem acolamento, causando regurgitação de grau importante. A dilatação aneurismática do átrio direito apresentava-se com área de 146 cm^2^ (102 cm/m^2^). O átrio esquerdo tinha dimensão normal. A área funcional do ventrículo direito era de 15 cm^2^ com disfunção sistólica de grau discreto à análise qualitativa. Ventrículo esquerdo era normal. Regurgitação valvar pulmonar de grau discreto e valva aórtica trivalvular sem disfunção. Artérias pulmonares confluentes com arco aórtico normal à esquerda. Na caracterização diagnóstica concluiu-se pela dilatação aneurismática do átrio direito e alterações da valva tricúspide com insuficiência tricúspide importante, secundárias à dilatação do anel valvar, afastando-se a impressão prévia de anomalia de Ebstein. A disfunção do ventrículo direito foi notada ser discreta ( Figura 2 ).

**Holter:** O ritmo de base era de taquicardia atrial com condução atrioventricular variável. A frequência cardíaca variou de 64 a 147 com média de 84 bpm. A condução ventricular (0,09) não mostrou alterações significativas. Não foram notadas arritmias ventriculares.

**Figura 3:**
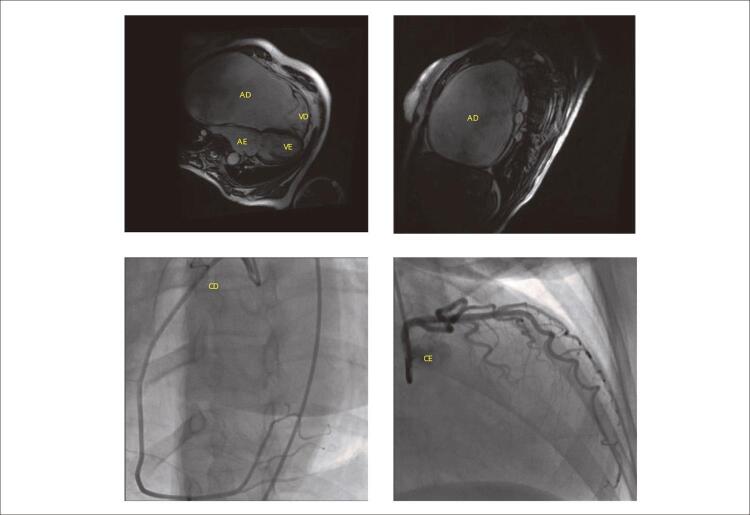
– RNM mostra o grande contraste da dilatação aneurismática do átrio direito em relação às demais cavidades que parecem até hipoplásicas e em decorrência, na cinecoronariografia as artérias coronárias se mostram alteradas, estando a coronária direita nitidamente alongada e a coronária esquerda desviada acentuadamente para a esquerda. VD: ventrículo direito; VE: ventrículo esquerdo; AD: átrio direito; AE: átrio esquerdo; CD: artéria coronária direita; CE: artéria coronária esquerda.

**Ressonância nuclear magnética:** mostrou acentuado aumento do átrio direito com diâmetro de 12,5 x 16,3 cm, sem trombos. A valva tricúspide se apresentava com implante valvar mais rebaixado, com acentuada insuficiência tricúspide. A função biventricular era normal e o ventrículo direito era menor, dada a acentuada atrialização ventricular ( Figura 3 ).

**Cateterismo cardíaco e Angiocardiografia:** o diagnóstico de insuficiência tricúspide foi confirmado com medidas semelhantes às do ecocardiograma, estando o átrio direito desproporcionalmente aumentado. A função biventricular era normal. As pressões correspondiam a: AD: 13, VD: 35/13, TP: 35/15-22, CP: 15, AE: 15, VE: 165/15, Ao: 165/75-105, RVP: 1,7W, RVS: 26,2 W, QP=QS: 3,5 l/min. As artérias coronárias se mostram alteradas estando a coronária direita muito alongada e a coronária esquerda desviada para a esquerda ( Figura 3 ).

**Diagnóstico clínico:** dilatação aneurismática do átrio direito com insuficiência tricúspide acentuada por dilatação do anel tricúspide e com disfunção discreta do ventrículo direito. Sintomas foram iniciados há 19 anos com arritmias supraventriculares.

**Conduta:** Em face da boa função ventricular direita foi orientada a conduta cirúrgica. Por esternotomia mediana verificada a grande dilatação aneurismática do átrio direito, que ocupava grande parte do hemitórax direito, deslocando a massa ventricular para a esquerda. Após instalação da CEC, com esfriamento a 30°C e cardioplegia sanguínea, foi aberto o átrio direito, e procedeu-se a ressecção de grande parte da parede livre desta cavidade (desde o sulco atrioventricular direito até próximo ao septo interatrial). Observou-se valva tricúspide muito displásica, não Ebstein, pois não havia acolamento de nenhum dos folhetos da valva tricúspide, e nem definição de alguma porção atrializada do ventrículo direito. Havia dilatação do ventrículo direito, com paredes finas e aparente redução da sua cavidade. Não foi possível estabelecer a adequada definição do anel valvar tricúspide. Realizada plástica da valva tricúspide, com redução do anel através de duas plásticas de *DeVega* . Após abertura do septo interatrial, notou-se valva mitral sem alterações.

Após fechamento do septo interatrial e do átrio direito observou-se redução muito importante do seu tamanho anterior. Tempo de CEC foi de 1:30 h e tempo de pinçamento de 60 minutos.

Reoperada após dois dias para retirada de grande quantidade de coágulos na loja pericárdica assim como da pleura direita. Realizada nesta ocasião ressecção parcial do pericárdio, além de plicatura e redução da dimensão do pericárdio, com consequente redução do espaço pericárdico.

Eletrocardiograma mostrou restituição do ritmo sinusal com frequência de 86 bpm, aumento discreto da amplitude dos complexos ventriculares, com bloqueio completo do ramo direito com complexo QRS de 0,13. A radiografia de tórax salientava diminuição nítida da área cardíaca.

Ecocardiograma no 18^o^ dia de pós-operatório mostrou veia cava inferior de 13mm e átrio direito com dilatação de grau importante. Plastia tricúspide sem gradiente residual significativo (máximo de 6mmHg e médio de 3,4mmHg) e refluxo de grau discreto a moderado. PSVD estimada em 28 mmHg. Insuficiência mitral de grau discreto a moderado (2 jatos). Ventrículo direito com dilatação de grau moderado. Função sistólica deprimida em grau discreto, à custa de hipocinesia apical em análise qualitativa. Disfunção diastólica tipo alteração de relaxamento ao Doppler tecidual. Ventrículo esquerdo com função sistólica preservada e disfunção diastólica tipo alteração de relaxamento ao Doppler convencional. Valvas pulmonar e aórtica com insuficiência de grau mínimo. Derrame pericárdico laminar.

Alta hospitalar obtida no 40^o^ dia pós-operatório com Captopril 12,5 mg, Digoxina 0,25mg, Espironolactona 25mg, Furosemida 20mg e Varfarina 5mg.

Na evolução a longo prazo, oito anos após a correção cirúrgica, observou-se boa evolução, em CF-I com cansaço a grandes esforços, com sopro sistólico ++/6 de intensidade na área tricúspide, e sem hepatomegalia. Estava em uso de losartana, varfarina e sinvastatina.

Eletrocardiograma mostrou a continuidade do ritmo sinusal com bloqueio do ramo direito com QRS de 0,13 de duração, complexos de maior voltagem e normalização da repolarização ventricular ( Figura 1 ).

Radiografia de tórax salientou aumento da área cardíaca à custa do arco atrial direito com índice cardiotorácico de 0,60. A trama vascular pulmonar era normal ( Figura 1 ).

Ecocardiograma mostrou: VD:43, VE:51, FEVE 67%. Plastia tricúspide com gradiente diastólico residual máximo de 4 mmHg e médio de 1 mmHg. Regurgitação tricúspide de grau importante (2 jatos), por falha de coaptação central. PSVD estimada em 39 mmHg. Átrio direito com dimensão aumentada em grau acentuado, com volume de 127 ml/m^2^. Ventrículo direito com dilatação moderada. Parede anterior media 5 mm. Via de saída dilatada medindo 45 mm. Função sistólica preservada à análise qualitativa, confirmada por TAPSE 1,7cm e FAC 40%. *“Strain* ” longitudinal de pico sistólico: 25%. Função diastólica preservada.

Gated mostrou funções ventriculares preservadas com VE: 58% e VD: 43%.

Holter mostrou frequência cardíaca variando de 32 a 105bpm com média de 50 bpm. Havia 78 extrassístoles supraventriculares e 6 ventriculares em 24h ( Figura 2 ).

## Discussão

A boa evolução do caso descrito nos surpreendeu em face da magnitude da dilatação do átrio direito verificada, em todos os parâmetros analisados de evolução, na clínica e na cirurgia. A radiografia de tórax com cardiomegalia extrema com índice cardiotorácico de 0,90, como expressão da grande repercussão da dilatação cardíaca, contrastava com a boa função ventricular direita. Este elemento motivou a indicação cirúrgica o que resultou em um evento de sucesso para a paciente na quinta década da vida e com outras complicações como arritmias e muito sintomática. Tal evolução tem sido mostrada em outros casos semelhantes, em adultos e em crianças.^1 , 3 , 12^

No entendimento dessa patologia, estudo anátomo-patológico foi obtido em um paciente com 88 anos de idade, que faleceu com insuficiência cardíaca e pneumonia, sendo o mais longevo descrito na literatura.^4^ Verificou-se que o átrio direito e o anel tricúspide eram muito dilatados e a parede atrial extremamente fina.^4^ Neste paciente, mesmo com tanta repercussão, havia descrição de fibrilação atrial, que se iniciara com 75 anos de idade. Em outra descrição patológica em paciente com 69 anos, que faleceu por câncer de pâncreas, havia cardiomegalia constatada há 19 anos, mantendo-se mesmo assim assintomático cardiovascular. O estudo mostrou acentuada dilatação atrial com degeneração muscular com necrose e fibrose difusa.^5^ Acrescenta-se outra descrição anatômica em evolução natural com 75 anos de idade, com características semelhantes.^8^ Chama a atenção de que a dilatação atrial direita pode se manter isolada, sem a ocorrência de dilatação do anel e assim sem insuficiência tricúspide, o que predispõe, no entanto, a arritmia supraventricular e a trombo interatrial, como ocorreu mesmo em um jovem de 17 anos.^6^

Nesta patologia, é de interesse notar também o caráter familiar descrito em dois adultos. Nesta família, o irmão faleceu com 40 anos com fibrilação atrial por morte súbita e a irmã, padecia da mesma dilatação do átrio direito, com 45 anos de idade.^7^

Na literatura, havia ainda outra descrição familiar dessa patologia em dois irmãos, ambos com bloqueio atrioventricular total associado, o que levantou a possibilidade de haver por isso nova síndrome clínica.^9^

Há ainda a descrita associação familiar da mãe de 53 anos com dois dos quatro filhos, descritos todos com aneurisma do átrio direito e ainda associados ao prolapso da valva mitral.^10^

Interessa ainda notar a associação familiar de dilatação de ambos os átrios, em relação à normalidade das outras estruturas cardíacas.^11^

As variantes anatômicas descritas salientam alguma diversidade de apresentação dessa patologia, sem poder se caracterizar como novas síndromes clínicas.

Poucos casos têm sido operados na literatura em idades mais avançadas, passadas quatro ou cinco décadas da vida, como apresentado no caso em discussão. Assim, encontra-se também descrito na literatura um caso similar, operado com 45 anos de idade, após sintomas terem surgido há 1 ano, tendo também mostrado boa evolução após ressecção parcial do átrio direito.^12^

Fica a recomendação nesta avaliação para que esses pacientes sejam corrigidos da dilatação isolada do átrio direito mais precocemente, ainda quando crianças e mesmo em pacientes assintomáticos, a se evitar o aparecimento de complicações que obscureçam a evolução posterior.
