# Supplementary material for: Dilatação Aneurismática Isolada do Átrio Direito, com Resolução Cirúrgica, em Adulta de 53 Anos
Source: Arq Bras Cardiol. 2023 Jun 7;120(6):e20220870. [Article in Portuguese] doi: 10.36660/abc.20220870 (PMC10263404; doi:10.36660/abc.20220870)
Supplement: Translation

# Translation: Isolated Aneurysmal Dilatation of the Right Atrium, with Surgical Resolution, in a 53-Year-Old Adult

**Authors:** Edmar Atik, Alessandra Costa Barreto, Maria Angélica Binotto, Marcelo B. Jatene

**Affiliations:** 1 Brazil Instituto do Coração do Hospital das Clínicas da Faculdade de Medicina da Universidade de São Paulo, São Paulo, SP – Brazil

**Keywords:** Congenital Abnormalities/surgery, Heart Aneurysm, Arrhythmias, Cardiac, Cardiomegaly, Ebstein Anomaly/surgery, Pericardial Effusion, Isolated Aneurysm of Right Atrium

## Introduction

Isolated aneurysmal dilatation of the right atrium is a rare congenital anomaly. Its identification occurs in several age groups, from the fetus to adulthood. The clinical manifestation of this anomaly varies according to the magnitude of the dilation, from asymptomatic patients, usually in children and young people, to the presence of difficult-to-control supraventricular arrhythmias, right heart failure, airway obstruction, pneumonia, and thromboembolic events in adults. The diagnosis is established after verification of cardiomegaly in routine radiographic examinations and confirmed later by echocardiogram. It is noteworthy in this pathology the disproportionate size of the right atrium concerning the other cardiac cavities, with the tricuspid ring also dilated but with a preserved tricuspid valve. The tricuspid insufficiency that accompanies the condition results from the dilation of the valve ring. The recommendation for surgical correction then becomes prophylactic in childhood, given the good evolution verified later.

The differential diagnosis includes Ebstein’s anomaly, pericardial effusion, and mediastinal thoracic tumors.

Antiarrhythmic treatment and antithrombotic medication, represented by ASA, usually initiate clinical management, given the presence of supraventricular arrhythmias and cavitary thrombi, which are more commonly found in adulthood.^1 - 12^ The atrial resection surgery, initially conceived by Morrow and Behrendt in 1968,^13^ was oriented to symptomatic patients. These authors confirmed the good surgical result in a 23-year-old woman with atrial flutter and cardiomegaly, with an aneurysm of the right atrium. A good evolution was observed in this patient, in sinus rhythm and without symptoms after the correction. Since that beginning, surgical management has been advocated, having even extended to asymptomatic patients, as an elective and preventive procedure for known complications.^1 , 2^ Its diagnosis during fetal life has guided elective surgical management in the first years of life.^2 , 3^ The long evolution described in some non-operated cases, with 69 and 88 years of age, does not generally express benignity, as observed in this disease with many evolutionary complications.^4 , 5^

Few cases have been operated on in older adulthood,^12^ which motivated us to demonstrate in this report that a female patient was operated on at 53.

### Case description

**Clinical data:** a 53-year-old patient had reported tachycardic palpitations for 19 years (from 34 years old) when using amiodarone, and diltiazem was started. For 3 years, progressive tiredness and swollen belly, with improvement after introducing spironolactone, furosemide, atenolol, and enalapril, in addition to warfarin and omeprazole.

At this time, the physical examination revealed good general condition, flushed, hydrated, acyanotic, Weight: 52.00 Kg, Height: 148 cm, BMI: 23.74, PA: 100/65 mm Hg, HR: 78 bpm, in rhythm irregular.

Precordium sharp impulses on the left external edge with a systolic murmur in the tricuspid area, 2 + / 6, rough, with irradiation to the right axillary line. The sounds were hypophonic.

Liver 3 cm from the right costal margin. Clean lungs.

### Complementary Exams

**Electrocardiogram:** irregular rhythm in atrial fibrillation, with a heart rate of 67 bpm, QRS complex at low voltage in the frontal plane with a maximum amplitude of 2 mm in all complexes. Low voltage in the derivations of the horizontal plane with complex Qs in V1, thickened S waves from V4 to V6, and complex Rs in V6. Diffuse changes in ventricular repolarization with negative T wave from V1 to V5. AQRS: -10o, and AT: 0o ( Figure 1 ).

**Figure 1:** – Chest X-rays and ECGs highlight the exaggeratedly enlarged cardiac area with low voltage of the QRS complex in the period prior to the surgery of aneurysmatic dilation of the right atrium on the left in contrast to the long-term postoperative findings on the right, with a decrease in cardiomegaly and an increase in electrical potentials.

**Chest radiography:** Marked increase in the cardiac area with right and left arches approaching the bilateral costal hip with a cardiothoracic index of 0.90. The pulmonary vascular network was reduced bilaterally ( Figure 1 ).

**Echocardiogram:** showed marked dilation of the right atrium in a great disproportion with the other cavities. The main measures corresponded to the mitral valve: 25 mm, tricuspid valve: 35, pulmonary valve: 21, aortic valve: 14, RPA: 21, LPA: 20, LA: 33, Ao: 28, S = PP: 7, EFLV: 58%.

The atrioventricular and ventricular-arterial connections were concordant. Patent Oval foramen with left-right shunt and the ventricular septum was intact. The tricuspid valve presented with an enlarged valve ring and a change in its disposition due to aneurysmatic dilation of the right atrium. The cusps were slightly thickened, with central coaptation failure and without padding, causing regurgitation of an important degree. Aneurysmatic dilation of the right atrium was 146 cm^2^(102 cm/m^2^). The left atrium was of normal size.

The functional area of the right ventricle was 15 cm^2^with mild systolic dysfunction on qualitative analysis. The left ventricle was normal. Pulmonary valve regurgitation of a mild degree and trivalvular aortic valve without dysfunction. Pulmonary arteries confluent with normal aortic arch on the left. In the diagnostic characterization, aneurysmal dilation of the right atrium and alterations of the tricuspid valve with major tricuspid insufficiency, secondary to the dilation of the valve ring, were concluded, removing the previous impression of Ebstein’s anomaly. The right ventricle dysfunction was noted to be mild ( Figure 2 ).

**Figure 2:** – Echocardiograms, in periods prior to the left and after surgery on the right, highlight the marked increase in the right atrium in sharp contrast to the other cardiac cavities in the presence of tricuspid regurgitation. On the right, these elements almost normalized in the images after the surgical intervention. RA: right atrium; LA: left atrium; RV: right ventricle; LV: left ventricle; TI: tricuspid insufficiency.

**Holter:** The baseline rhythm was atrial tachycardia with variable atrioventricular conduction. Heart rate ranged from 64 to 147, averaging 84 bpm. Ventricular conduction (0.09) did not show significant changes. No ventricular arrhythmias were noted.

**Nuclear magnetic resonance:** showed a marked increase in the right atrium with a diameter of 12.5 x 16.3 cm, without thrombi. The tricuspid valve presented with a lower valve implant with marked tricuspid insufficiency. The biventricular function was normal, and the right ventricle was smaller, given the marked ventricular atrialization ( Figure 3 ).

**Figure 3:** – MRI shows the great contrast of aneurysmal dilatation of the right atrium concerning the other cavities that seem even hypoplastic and, as a result, the altered coronary arteries, the right coronary being sharply elongated and the left coronary deviated sharply to the left. RA: right atrium; LA: left atrium; RV: right ventricle; LV: left ventricle; RCA: right coronary artery; LCA: left coronary artery.

**Cardiac catheterization and Angiocardiography:** the diagnosis of tricuspid insufficiency was confirmed with measurements similar to those of the echocardiogram, with the right atrium being disproportionately enlarged. The biventricular function was normal. The pressures corresponded to RA: 13, RV: 35/13, TP: 35 / 15-22, CP: 15, LA: 15, LV: 165/15, Ao: 165 / 75-105, PVR: 1.7W, SVR: 26.2 W, QP = QS: 3.5 l / min. The coronary arteries are altered, with the right coronary being very elongated and the left coronary deviated to the left ( Figure 3 ).

**Clinical diagnosis:** aneurysmatic dilation of the right atrium with marked tricuspid insufficiency due to dilation of the tricuspid ring and slight dysfunction of the right ventricle. Symptoms started 19 years ago with supraventricular arrhythmias.

**Conduct:** Surgical conduct was advised because of the good right ventricular function. The median sternotomy showed a large aneurysmal dilation of the right atrium, which occupied a large part of the right hemithorax, displacing the ventricular mass to the left. After CPB installation, with cooling to 30 ° C and blood cardioplegia, the right atrium was opened, and a large part of the free wall of this cavity was resected (from the right atrioventricular groove to close to the interatrial septum). A very dysplastic tricuspid valve was observed, not Ebstein, as there was no padding of any of the leaflets of the tricuspid valve nor the definition of any atrialized portion of the right ventricle. The right ventricle was dilated, with thin walls and an apparent reduction of its cavity. Establishing the proper definition of the tricuspid valve ring was impossible. The plastic of the tricuspid valve was performed, reducing the ring through two DeVega plastics. After opening the interatrial septum, a mitral valve was seen without changes.

After closing the interatrial septum and the right atrium, a very important reduction in its anterior size was observed. CPB time was 1:30 h, and clamping time was 60 minutes.

Reoperated after two days to remove a large number of clots in the pericardial store as well as the right pleura. On this occasion, partial resection of the pericardium was performed, in addition to plication and reduction of the size of the pericardium, with a consequent reduction of the pericardial space.

Electrocardiogram showed restitution of the sinus rhythm with a frequency of 86 bpm, a slight increase in the ventricular complexes’ amplitude, and a complete block of the right branch with a QRS complex of 0.13. The chest X-ray showed a clear decrease in the cardiac area.

Echocardiogram on the 18^th^postoperative day showed a 13mm inferior vena cava and right atrium with significant dilation. Tricuspid plasty without significant residual gradient (maximum of 6mmHg and average of 3.4mmHg) and mild to moderate reflux. RVSP is estimated at 28 mmHg. Mitral insufficiency of mild to moderate degree (2 jets). Right ventricle with moderate degree dilation. Discreetly depressed systolic function at the expense of apical hypokinesia in qualitative analysis.

Diastolic dysfunction type of relaxation alteration on tissue Doppler. Left ventricle with preserved systolic function and diastolic dysfunction like relaxation alteration on conventional Doppler. Pulmonary and aortic valves with minimal grade insufficiency. Laminar pericardial effusion.

Hospital discharge was obtained on the 40^th^postoperative day with Captopril 12.5 mg, Digoxin 0.25mg, Spironolactone 25mg, Furosemide 20mg, and Warfarin 5mg.

In the long-term evolution, eight years after the surgical correction, a good evolution was observed in FC-I with fatigue with great efforts, with systolic murmur ++/6 of intensity in the tricuspid area, and without hepatomegaly. He was using losartan, warfarin, and simvastatin.

Electrocardiogram showed continuity of sinus rhythm with right branch block with QRS of 0.13 in duration, higher voltage complexes, and normalization of ventricular repolarization ( Figure 1 ).

Chest radiography showed increased cardiac area at the expense of the right atrial arch with a cardiothoracic index of 0.60. The pulmonary vascular network was normal ( Figure 1 ).

Echocardiogram showed: RV: 43, LV: 51, LVEF 67%. Tricuspid plasty with a maximum residual diastolic gradient of 4 mmHg and a mean of 1 mmHg.

Major degree tricuspid regurgitation (2 jets) due to central coaptation failure. RVSP estimated at 39 mmHg. Right atrium with a markedly increased dimension, with a volume of 127 ml / m^2^. Right ventricle with moderate dilation. The anterior wall measured 5 mm. Dilated outlet track measuring 45 mm. Systolic function preserved on analysis qualitative, confirmed by TAPSE 1.7cm and FAC 40%. Systolic peak longitudinal strain: 25%. Preserved diastolic function.

Gated showed ventricular functions preserved with LV: 58% and RV: 43%.

Holter showed a heart rate ranging from 32 to 105 bpm, averaging 50 bpm. There were 78 supraventricular and 6 ventricular extrasystoles in 24 hours ( Figure 2 ).

## Discussion

The good evolution of the case described surprised us because the magnitude of the dilation of the right atrium verified, in all the analyzed parameters of evolution, in the clinic and the surgery. Chest radiography with extreme cardiomegaly with a cardiothoracic index of 0.90, as an expression of the great repercussion of cardiac dilation, contrasted with good right ventricular function. This element motivated the surgical indication, which resulted in a successful event for the patient in the fifth decade of life and with other complications such as arrhythmias and very symptomatic. Such evolution has been shown in similar cases in adults and children.^1 , 3 , 12^

To understand this pathology, an anatomopathological study was obtained on an 88-year-old patient, who died of heart failure and pneumonia, being the longest-lived described in the literature.^4^ It was found that the right atrium and the tricuspid ring were very dilated and the atrial wall extremely thin.^4^ In this patient, even with such repercussions, there was a description of atrial fibrillation, which had started at the age of 75 years. In another pathological description of a 69-year-old patient who died of pancreatic cancer, cardiomegaly was observed 19 years ago, although he remained asymptomatic cardiovascular. The study showed marked atrial dilation with muscle degeneration with necrosis and diffuse fibrosis.^5^ Another anatomical description in natural evolution is added at the age of 75, with similar characteristics.^8^ Notably, the right atrial dilation can remain isolated, without ring dilation and thus without tricuspid insufficiency, which predisposes to supraventricular arrhythmia and intraatrial thrombus, as occurred even in a 17-year-old young man.^6^

It is also interesting to note the familiar character described in two adults in this pathology. In this family, the brother died at the age of 40 from atrial fibrillation due to sudden death, and the sister suffered from the same dilation of the right atrium at the age of 45.^7^

In the literature, there was yet another family description of this pathology in two brothers, both with associated total atrioventricular block, which raised the possibility of having a new clinical syndrome for this reason.^9^

There is also the described family association of the 53-year-old mother with two of the four children, all of whom were described as having an aneurysm of the right atrium and still associated with mitral valve prolapse.^10^

It is also interesting to note the familial association of dilation of both atria concerning the normality of the other cardiac structures.^11^

The anatomical variants described highlight some diversity in the presentation of this pathology without being able to be characterized as new clinical syndromes.

Few cases have been operated on in the literature at older ages, four or five decades afterlife, as presented in the case under discussion. Thus, a similar case, operated on at 45 years of age, after symptoms having appeared 1 year ago, has also been described in the literature, showing good evolution after partial resection of the right atrium.^12^

It is recommended in this evaluation that these patients be corrected from isolated dilation of the right atrium earlier, even as children and even in asymptomatic patients, to avoid the appearance of complications that obscure the subsequent evolution.
